# WRKY Transcription Factors: Molecular Regulation and Stress Responses in Plants

**DOI:** 10.3389/fpls.2016.00760

**Published:** 2016-06-03

**Authors:** Ujjal J. Phukan, Gajendra S. Jeena, Rakesh K. Shukla

**Affiliations:** Biotechnology Division, Central Institute of Medicinal and Aromatic PlantsLucknow, India

**Keywords:** WRKY, multiple response, proteasome-mediated degradation, retrograde signaling

## Abstract

Plants in their natural habitat have to face multiple stresses simultaneously. Evolutionary adaptation of developmental, physiological, and biochemical parameters give advantage over a single window of stress but not multiple. On the other hand transcription factors like WRKY can regulate diverse responses through a complicated network of genes. So molecular orchestration of WRKYs in plant may provide the most anticipated outcome of simultaneous multiple responses. Activation or repression through W-box and W-box like sequences is regulated at transcriptional, translational, and domain level. Because of the tight regulation involved in specific recognition and binding of WRKYs to downstream promoters, they have become promising candidate for crop improvement. Epigenetic, retrograde and proteasome mediated regulation enable WRKYs to attain the dynamic cellular homeostatic reprograming. Overexpression of several WRKYs face the paradox of having several beneficial affects but with some unwanted traits. These overexpression-associated undesirable phenotypes need to be identified and removed for proper growth, development and yeild. Taken together, we have highlighted the diverse regulation and multiple stress response of WRKYs in plants along with the future prospects in this field of research.

## Introduction

Environmental fluctuations consisting of abiotic and biotic stresses impart detrimental effect on economically important plants. Evolutionary alterations helped the plants to adapt under these adverse conditions. Some genus, rather species or varieties show higher tolerance level to these stresses than others (Phukan et al., 2014). This variation is regulated through a wide network of transcriptional and hormonal crosstalk. The response to external hazardous stimuli is percieved by signal molecules which induce primary genes associated with the stress. Subset of these genes include many transcription factors (TFs) like WRKY, ERF, NAC, and MADS. WRKYs are of particular interest as they are involved in diverse biotic/abiotic stress responses as well as in developmental/physiological processes (Jiang et al., 2015). They recognize the W-box present in the promoter of target genes and induce their expression to achieve cellular homeostsis. In this review we will mainly emphasize the importance of WRKYs in regulating various plant processes including rarely discussed topics like proteasome-mediated degradation, epigenetic regulation, and retrograde signaling. We will highlight their mode of action, phosphorylation properties, also interaction at both protein and DNA level. In previous studies the focus was mainly on the roles of WRKYs in regulation of stress and development. Although stress and developmental response would be considered but we will highlight the recent progress made in this area to the best of our knowledge. Our aim is to make a comprehensive review to answer how a single TF can regulate various contrasting responses.

## WRKYs: Classification and Functional Domains

WRKY is a major TF family of plants though there are reports of WRKY in soil-living amoeba like *Dictyostelium discoideum* and flagellated protozoan like *Giardia lamblia.* Large numbers of WRKY are found in plants like 109 in rice and 74 in *Arabidopsis*. They contain ≈60 amino acid long four-stranded β-sheet WRKY DNA binding domain/s (DBD) and Zinc-finger motifs. Based on these they are divided into group I (2 WRKY DBDs), II (single DBD with different C2H2 zinc finger), and III (single DBD with C2HC zinc finger). Group II that is not monophyletic is divided into IIa, IIb, IIc, IId, and IIe based on the primary amino acid sequence (Rushton et al., 2010). Additionally they contain basic nuclear localization domain, leucine zippers, serine-threonine-rich region, glutamine-rich region, proline-rich region, kinase domain, and TIR-NBS-LRR domain (Chen et al., 2012). A Calmodulin (CaM)-binding domain (DxxVxKFKxVISLLxxxR) is also observed in *Arabidopsis* Group IId WRKYs like AtWRKY7 (Park et al., 2005). The primary WRKYGQK motif of DBD shows some anomaly like WRRY, WSKY, WKRY, WVKY, or WKKY (Xie et al., 2005). WRKY TFs interact with W-box (with core motif TTGACC/T) and clustered W-boxes present in the promoter of downstream genes to regulate the dynamic web of signaling through kinase or other phosphorylation cascades. Although WRKYs bind specifically to W- box there are reports of them binding to non-W box elements like OsWRKY13 binds to both PRE4 element (TGCGCTT) and W box (Cai et al., 2008). HvWRKY46 (SUSIBA2) can bind to both W box and a sugar-responsive (SURE) element – TAAAGATTACTAATAGGAA (Sun et al., 2003). In contrast NtWRKY12 can bind to a SURE-like element but not to the W box. NtWRKY12 has the sequence WRKYGKK instead of WRKYGQK and binds specifically to the WK box – TTTTCCAC (Sun et al., 2003). WRKY DBD is mostly conserved and it interacts mainly with W-box *cis* motif, though the activation of downstream genes under a particular condition is very specific. This might be because of the motifs and domains outside of DBD that provides binding specificity to WRKY TFs under different conditions. Based on their class and amino acid sequence it is observed that β1 and β2 sheets of DBD are mostly conserved while β3 and β4 sheets shows discripancy either in terms of number of amino acids or conservation. Therefore nature of binding affinity of different groups of WRKY to W-box and others seems to be ambiguous, which needs further study and exploration of the domains present outside of DBD.

## WRKYs in Multiple Responses

**FIGURE 1:**
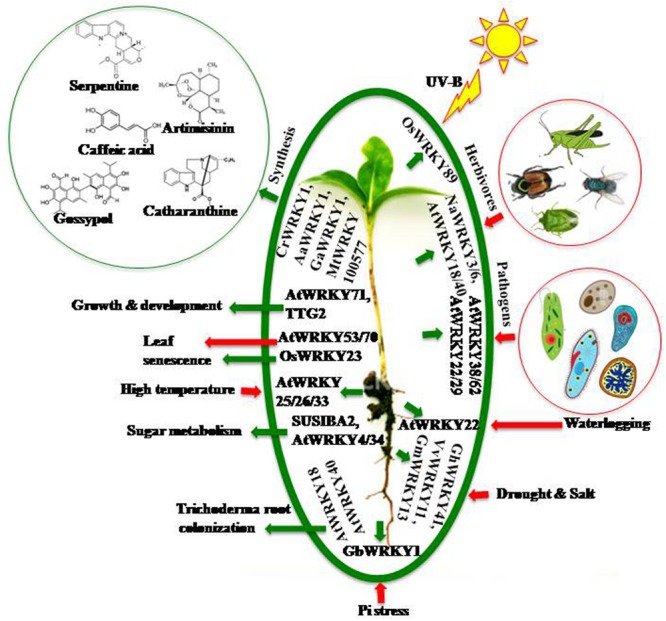
**Multiple role of WRKYs under different environmental conditions.** In response to multiple stimuli plants recruit various WRKYs to regulate downstream cascade. The green arrows indicate a positive or beneficial regulation while red arrows indicate negative or harmful regulation. In response to different stresses WRKYs provide tolerance or resistance to the respective plants; like AtWRKY29, 38, 62 provide resistance to pathogen attack; AtWRKY3, 6, 18, 40 provide resistance to herbivores; OsWRKY89 protects plant from harmfull UV radiation; many WRKYs impart drought and salt tolerance; AtWRKY22 provides waterlogging tolerance; GbWRKY1 helps plant in phosphate starvation and AtWRKY25, 26 provide thermotolerance. They are also involved in metabolic and developmental responses; like CrWRKY1, AaWRKY1, GaWRKY1 are involved in secondary metabolism; AtWRKY71, TTG2 are involved in growth and development; SUSIBA2, AtWRKY34 are involved in sugar metabolism and AtWRKY18, 40 are involved in trichoderma colonization. AtWRKY53, 70 induce leaf senescence while OsWRKY23 prevents it.

WRKYs act through various interconnecting networks to regulate multiple responses simultaneously whether it is biotic, abiotic, or physiological (Banerjee and Roychoudhury, 2015) (**Figure 1**).

### Biotic Stress

If we consider biotic stress several WRKYs are able to confer resistance towards multiple bacterial or fungal agents. AtWRKY52 containing a TIR–NBS–LRR (Toll/interleukin-1 receptor–nucleotide-binding site-leucine-rich repeat) domain acts together with RPS4 to provide resistance against fungal pathogen *Colletotrichum higginsianum* and bacterial pathogen *Pseudomonas syringae* (Narusaka et al., 2009). AtWRKY52 also shows nuclear interaction with the bacterial effector PopP2 and provides immunity to bacterial pathogen *Ralstonia solanacearum* (Deslandes et al., 2002). NBS–LRR–WRKY interaction is also seen in AtWRKY52/RRS1, AtWRKY16/TTR1, and AtWRKY19, which helps in the activation of defense related genes (Rushton et al., 2010). AtWRKY16 and AtWRKY19 also possesses similar TIR–NBS–LRR domain suggesting the involvement of these proteins in defense related ETI pathway (Chi et al., 2013). AtWRKY50/51 mediates SA- and low oleic acid- dependent repression of JA signaling, resulting in enhanced resistance to *Alternaria brassicicola* but increased susceptibility to *Botrytis cinerea* (Gao et al., 2011). AtWRKY1 binds to its own promoter and acts as an activator of fungal elicitor-induced gene *PcPR1–1* in parsley (Turck et al., 2004). PtrWRKY73 is involved in diseases resistance in *Arabidopsis* (Duan et al., 2015). CC-NB-LRR (coiled coil-nucleotide-binding site-leucine-rich repeat) protein Pb1 (Panicle blast 1) confers broad-spectrum resistance to *Magnaporthe oryzae* by physically interacting with OsWRKY45 (Inoue et al., 2013). This property of a single TF to provide multiple resistance against biotic agents can be targeted for improved variety development.

### Abiotic Stress

Similarly a single WRKY can mediate several abiotic responses. OsWRKY74 modulates Pi homeostasis, Fe starvation, and cold stress in rice (Dai et al., 2016). AtWRKY71 on one side accelerates flowering by regulating FLOWERING LOCUS T and LEAFY while on the other side regulates shoot branching by activating RAX genes (Guo et al., 2015; Yu et al., 2016). GhWRKY41/SpWRKY1 enhances salt and drought tolerance in transgenic tobacco by regulating stomatal conductance and ROS levels (Chu et al., 2015; Li J.B. et al., 2015). FcWRKY70 is involved in drought tolerance and putrescine synthesis (Gong et al., 2015). GbWRKY2 (from *Ginkgo biloba*), PgWRKY1 (from *Panax ginseng*), and SiWRKY066/082 (from *Setaria italica*) are involved in stress and hormone signaling (Liao et al., 2015; Muthamilarasan et al., 2015; Nuruzzaman et al., 2016). AtWRKY46 regulates development, stress and hormonal response by facilitating growth of lateral roots in osmotic/salt stress through ABA signaling and auxin homeostasis (Ding et al., 2015). HaWRKY76 from sunflower confers drought and flood tolerance in transgenic *Arabidopsis* (Raineri et al., 2015a). GmWRKY13/54 are involved in drought and salt tolerance (Zhou et al., 2008). BhWRKY1 binds to *BhGolS1* promoter involved in providing drought and cold tolerance (Wang et al., 2009). PsWRKY from *Papaver somniferum* is shown to be induced by various treatments like wounding, cold, salt, ABA, drought as well as MeJA and regulate Benzylisoquinoline Pathway (Mishra et al., 2013). GbWRKY1 in transgenic *Arabidopsis* led to enhanced auxin sensitivity and resulted in attenuated Pi starvation stress symptoms like reduced accumulation of pigments mainly anthocyanin and impaired density of lateral roots (Xu et al., 2012). Many *Arabidopsis* WRKYs (6, 16, 18, 19, 27, 32, and 40) regulate diverse cellular functions by physically interacting with 14-3-3 proteins (Chang et al., 2009). Many WRKYs like AtWRKY33 *via* its C-terminal domain interact with multiple VQ proteins (with VQ-related motif -FxxxVQxLTG) including SIB1 and SIB2 (Sigma Factor-Interacting Protein) to regulate multiple abiotic stresses (Lai et al., 2011; Wang M. et al., 2015). Crop loss in climate challenging regions could be averted if transgenic approach of crop improve ment is applied to diverse economically important plants. Therefore WRKYs are of prime importance as they can regulate multiple abiotic stresses simultaneously.

### Biotic and Abiotic Stress

There are some WRKYs which regulate both biotic as well as abiotic responses. *Gossypium hirsutum* WRKY25 (GhWRKY25) negatively regulates drought stress and *B. cinerea* infection but positively regulates salt stress in transgenic tobacco (Liu et al., 2015). Overexpression of GhWRKY27a reduces tolerance to drought and resistance to *Rhizoctonia solani* infection in transgenic tobacco (Yan et al., 2015). VvWRKY1 induces expression of JA pathway-related genes and confers higher tolerance to the downy mildew and provides tolerance to osmotic stress in *Vitis* (Liu et al., 2011; Marchive et al., 2013). VvWRKY11 also regulates drought tolerance in *Arabidopsis* (Liu et al., 2011). In pepper CaWRKY40 is regulated by CaWRKY6, which in turn regulates *R. solanacearum* resistance, also provides tolerance towards high-temperature and high-humidity (Cai et al., 2015). Growth and yeild of plants are severely affected by the stagnant waterlogged or submerged condition (Phukan et al., 2015). AtWRKY22 provides submergence tolerance by interacting with the ACS7 promoter and activating downstream ethylene signaling (Hsu et al., 2013). AtWRKY22 also regulates dark induced leaf senescence, promotes susceptibility to aphids, modulates salicylic acid and jasmonic acid signaling (Zhou et al., 2011; Kloth et al., 2016). If a plant can withstand multiple biotic as well as abiotic stresses without compromising growth and yield in field condition, agricultural revolution could be attained. By manipulating expression of a single TF like WRKY this multiple tolerance trait could be developed. For this purpose there is a necessity to understand the proper structural and functional relationship of these multiple regulatory WRKY TFs. Further exploration of their function in contrasting verities against stress treatments will also help us to develop plants which may naturally sustain themselves under multiple stress response.

### Secondary Metabolism

Secondary metabolites are specialized plant products that are associated with a broad assortment of biological functions. WRKY TFs are shown to regulate production of several secondary metabolites like phenolic compounds along with lignin, flavanols, and tannins (Guillaumie et al., 2010; Wang et al., 2010). AtWRKY23 in *Arabidopsis* regulates the production of flavanols in auxin inducible manner (Grunewald et al., 2012). Another important subset of tannin compounds, proanthocyanin is regulated by AtWRKY44 (TRANSPARENT TESTA GLABRA2) (Johnson et al., 2002). MYB-bHLH-WD40 controls the expression of AtWRKY44 that is a key regulator of anthocyanin production indicating the crosstalk of WRKY TFs with other networks regulating specialized metabolism (Ishida et al., 2007). WRKYs also act as a key regulator of alkaloid biosynthesis. In *Catharanthus* species 25% of WRKYs are induced in response to jasmonate and could potentially regulate terpene indole alkaloid biosynthetic genes (Schluttenhofer et al., 2014). In *Catharanthus roseus* CrWRKY1 regulate the expression of TRYTOPHAN DECARBOXYLASE that is involved in the synthesis of indolic tryptamine precursors (Suttipanta et al., 2011). Additionally, TIA pathway metabolites such as catharanthine and serpentine accumulates differentially in CrWRKY1 RNAi lines of hairy root cultures, suggesting that CrWRKY1 regulates the metabolic flux by regulating the genes within the pathway. CjWRKY1 from *Coptis japonica* governs the expression of berberine biosynthetic gene without affecting the primary metabolism (Kato-Noguchi et al., 2008). Similarly, benzylisoquinoline alkaloids (BIAs) are also regulated by WRKY TFs. Wound induced PsWRKY may substantially regulate BIA pathway as it interacts *in vitro* with the W-box cis-elements present in the promoter of seven transcripts involved in the pathway (Mishra et al., 2013). Overexpression of AtWRKY1 in *Eschscholzia californica* accumulates sanguinarine and chelirubine (Apuya et al., 2008). GaWRKY from *Gossypium arboreum* regulates sesquiterpene cyclase at a pathway branch point enhancing the production of gossypol, an antifeedant phytoalexin (Xu et al., 2004). *Solanum lycopersicum* SlWRKY71 is involved in the activation of three monoterpene synthase genes, suggesting multiple simultaneous regulation by a single WRKY TF (Spyropoulou et al., 2014). Biosynthesis of antimalarial drug artimisinin produced in trichomes of *Artimisia annua* is regulated by AaWRKY1 (Ma et al., 2009). TcWRKY1 from *Taxus chinensis* regulates the expression of rate limiting gene DBAT (10-deacetylbaccatin III-10 β-O-acetyl transferase) involved in the biosynthesis of anticancer drug taxol (Li et al., 2013). Rice produces terpenes for defense against pathogens and herbivores. OsWRKY45 found to regulate the production of diterpenoid phytoalexin like momilactone, phytocassane and oryzalexin by priming the expression of biosynthetic genes (Akagi et al., 2014). Rice OsWRKY76 activates cold stress tolerance but suppresses PR genes and production of phytoalexins like terpene and phenylpropanoid sakuranetin (Yokotani et al., 2013). PqWRKY1 from *Panax quinquefolius*, is associated with increased accumulation of ginsenosides, a group of triterpene compounds (Sun et al., 2013). Ectopic expression of PqWRKY1 in *Arabidopsis* up-regulates genes involved in triterpene biosynthesis, indicating that WRKYs are capable of regulating metabolic pathways in other species. The expression of HbWRKY1 has been associated with increased biosynthesis of natural rubber, a polyisoprenoid derived from wounding the bark of the tropical tree *Hevea brasiliensis* (Zhang et al., 2012). So directly or indirectly WRKYs regulate plant defense response and development by altering/enhancing secondary metabolite biosynthesis. So WRKY based elicitor/stimuli responsive over expression systems should be developed for exploiting the regulatory role of these TFs on secondary metabolism.

## Crosstalk of WRKYs in Multiple Responses

The regulation of multiple responses includes a huge interconnecting network and interactions. Therefore it is observed that many WRKY TFs work in cluster to mediate various responses in stress tolerance and development.

### AtWRKY18–40–60 Cluster

AtWRKY18 stimulates SA-signaling and enhances resistance to *P. syringae* while its coexpression with AtWRKY40 or AtWRKY60 enhances their susceptible (Xu et al., 2006). AtWRKY18 and AtWRKY60 also enhance plant sensitivity to salt and osmotic stress while AtWRKY40 antagonizes this effect (Chen et al., 2010). The three WRKY proteins form both homocomplexes and heterocomplexes through Leu zipper motif. AtWRKY60-18 interaction increases DNA binding ability of AtWRKY18 while AtWRKY60-40 interaction decreases DNA binding ability of AtWRKY40 (Xu et al., 2006). AtWRKY18 and AtWRKY40 recognize a cluster of W-box sequences in the *AtWRKY60* promoter to probably activate ABA signaling (Geilen and Böhmer, 2015). 14-3-3 proteins also interact and phosphorylate AtWRKY18 and AtWRKY40 to regulate ABA and stress-activated signaling (Shen et al., 2003; Shang et al., 2010). Also in excess of ABA Mg-chelatase carrying an ABA receptor interacts and represses AtWRKY18, AtWRKY40, and AtWRKY60 (Shang et al., 2010). AtWRKY18, AtWRKY40, and AtWRKY60 are involved in transcriptional regulation of *ABFs/AREBs* by binding to the W-box element present in their promoters (Antoni et al., 2011). Also AtWRKY18 and AtWRKY40 both stimulates JA-signaling via suppression of JAZ repressors and negatively regulates the expression of the defense genes *FMO1, PAD3, and CYP71A13*, finally leading to the enhanced *Trichoderma* root colonization (Brotman et al., 2013). *Trichoderma* spp. stimulates plant growth and resistance to a wide range of adverse environmental conditions. AtWRKY18 and AtWRKY40 also negatively regulates *Golovinomyces orontii* infection (Schön et al., 2013). PtrWRKY40 from *Populus trichocarpa* shows similarity with AtWRKY18/40/60 and have a negative role in resistance to *Dothiorella gregaria* infection in poplar but acts as a positive regulator of resistance toward the *B. cinerea* in *Arabidopsis* (Karim et al., 2015). These interactions and crosstalk does not limit within this cluster but extend to downstream cascades to regulate multiple responses. To identify the entire web of interaction, large-scale studies should be carried out on regulatory networks.

### OsWRKY45 Cluster

OsWRKY45-1/2 are involved in the basal defense response in rice. The regulation is also differentially modulated like OsWRKY45-1 negatively regulates *X. oryzae* response while OsWRKY45-2 is a positive regulator of plant responses to *X. oryzae* (Tao et al., 2009). OsWRKY45-1 modulates SA and JA levels, while OsWRKY45-2 modulates only JA levels. OsWRKY45s also negatively regulate ABA response and provide enhanced salt and drought tolerance (Xie et al., 2005; Qiu and Yu, 2009). Cluster of OsWRKY45-2, OsWRKY13, OsWRKY42 is required for development of resistance to fungal pathogen *M. oryzae* in rice (Cheng et al., 2015). *In vivo* and *in vitro* DNA-protein as well as protein-protein interaction studies would be helpful to explore the integrating network involved. With high throughput bioinformatics approaches genome wide interactions should be studied for proper understanding of the regulatory mechanisms.

### OsWRKY24–51–71 Cluster

Another WRKY cluster present in rice is OsWRKY51/71 that represses *RAmy1A* α-amylase and thus regulates crosstalk of GA and ABA signaling in embryos. These two are ABA inducible which physically interacts in the nucleus and promotes the binding of OsWRKY71 to the *Amy32b* (GAMYB) promoter. Even though OsWRKY51 itself does not bind, it leads to the supression of GA inducible GAMYB through this interaction. When GA level increases, it induces the expression of GAMYB and inhibits OsWRKY51 and OsWRKY71, finally inducing expression of α-amylase (Zhang et al., 2004; Xie et al., 2006). OsWRKY24 also negatively regulates GA and ABA signaling though OsWRKY51/71 possess single DBD while OsWRKY24 possess two DBD. OsWRKY24 too represses the expression of *Amy32b* and *HVA22* to regulate several developmental responses (Zhang et al., 2009). Along with them OsWRKY53 and OsWRKY70 acts as negative transcriptional regulators of GA and ABA signaling (Zhang et al., 2015). Rice cultivation is widespread and so thus agents that affects its productivity. Therefore it is very important to identify the factors that may provide tolerance to these environmental stresses without compromising the yeild.

### Other Clusters

AtWRKY53 and AtWRKY70 negatively regulate leaf senescence in *Arabidopsis* (Miao et al., 2004; Ulker et al., 2007). AtWRKY53 also physically interacts with AtWRKY30 to control senescence progression by regulating ROS level (Besseau et al., 2012). Cross regulation among AtWRKY25, AtWRKY26 and AtWRKY33 is necessary for promoting plant thermo-tolerance (Li et al., 2011). ThWRKY4 from *Tamarix hispida* can form both homo- and hetero-dimers with ThWRKY2 and ThWRKY3 to mediate various abiotic responses (Wang L. et al., 2015). GmWRKY27 physically interacts with GmMYB174 to suppress expression of GmNAC29 under different stresses to induce tolerance in soybean plants (Wang F. et al., 2015). MaNAC5 from banana physically interacts with MaWRKY1/2 and cooperatively regulates defense response (Shan et al., 2016). HvWRKY38/1 both act as repressors of seed germination (Xie et al., 2007). HvWRKY38 is also involved in cold and drought response and its close homolog HvWRKY1 is involved in repression of basal defense. (Mare et al., 2004; Shen et al., 2007; Zou et al., 2008). LtWRKY21 from *Larrea tridentate* binds to the promoter of *HVA22* an ABA-responsive gene to regulate multiple stress responses. During abiotic stresses LtWRKY21, VP1, and ABI5 interacts to regulate downstream of ABI1 in ABA-mediated response cascade (Zou et al., 2004). *Arabidopsis* and rice are model plants and lots of studies have already been done but non-model economically important plants are still susceptible to climatic aberrations. So equal importance should be given to identify the crosstalks and regulatory responses of these plants under different conditions.

## Regulation of WRKYs at Different Levels

### Regulation at Transcriptional or Post-Transcriptional Level

**FIGURE 2:**
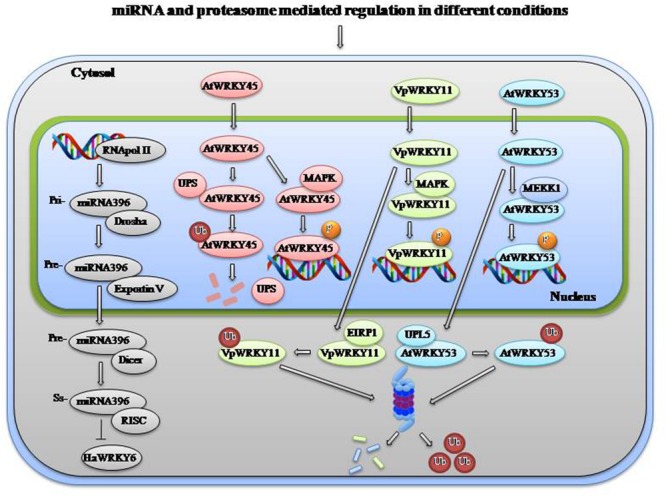
**miRNA and proteasome mediated degradation of WRKYs in different conditions.** Under normal condition, stress responsive WRKYs are tightly regulated by various mechanisms and after getting stress stimulus they are phosphorylated/activated by different mechanisms to regulate downstream genes. miRNA either lead to degradation or translational inhibition of WRKYs to regulate unwanted expression. An example of regulation/degradation of HaWRKY6 by miR396 cascade is shown. Also proteasome mediated degradation of AtWRKY45, VpWRKY11, and AtWRKY53 by UPS, EIRP1, and UPL5 has been shown, respectively. Single color has been given to all the factors involved in a paricular cascade. After onset of stress these WRKYs are phosphorylated by different MAPKs and they in turn regulate expression of downstream stress responsive genes. Abbreviations: UPL5: Ubiquitin protein ligase 5, UPS: nuclear ubiquitin-proteasome system, EIRP1: Erysiphe necator-induced RING finger protein 1, MAPK, Mitogen-activated protein kinase; Ub, Ubiquitin; RISC, RNA isolated silencing complex.

To obtain an accurate balance in stress and developmental responses, expression of WRKYs and their downstream activation is tightly regulated. For activation of certain WRKYs under biotic stress, ETI (Effector-triggered immunity) mediated regulation is required. HvWRKY1/2 are activated when fungal avirulence AVR10 effector is recognized by resistance protein MLA (mildew-resistance locus A) in the cytoplasm and the subsequent association of MLA with the WRKYs in the nucleus (Shen et al., 2007). *Nicotiana attenuate* NaWRKY3 is required for NaWRKY6 activation by fatty acid–amino conjugates found in the oral secretions of *Manducasexta* larva. After wounding mediated activation they process responses to herbivory (Skibbe et al., 2008). In another case of herbivory, *Spodoptera littoralis* induces the synthesis of JA-isoleucine that binds to a complex of receptor COI1 and repressor JAZ finally activating AtWRKY18 and AtWRKY40 (Schweizer et al., 2013). Similarly AtWRKY23 is upregulated upon *Heterodera schachtii* nematode infection (Grunewald et al., 2008). OsBWMK1 phosphorylates OsWRKY33, which binds to the W box element present in the promoter of several PR genes (Koo et al., 2009). PAD4, a key regulator of SA signaling regulates AtWRKY33 which provides resistance to *B. cinerea* and regulates genes involved in redox homeostasis, SA signaling, ET-JA-mediated cross-communication and camalexin biosynthesis (Qiu et al., 2008). BHLH and R2R3MYB TFs (WEREWOLF, GLABRA1, and TRANSPARENT TESTA) regulate the expression of TTG2 that plays a role in trichome and seed development by regulating expression of GLABRA2 (Ishida et al., 2007). TTG2 also increases sensitivity to salt stress through suppression of auxin biosynthetic genes (Li Q. et al., 2015). Zinc-finger protein Zat12 is induced by drought, osmotic, salinity, temperature, oxidative stress, and wounding which in turn transcriptionally regulates AtWRKY25 (Davletova et al., 2005; Mittler et al., 2006). Auxin regulates the expression of Auxin response factors (ARF7 and ARF19), which controls proper growth and development of root by regulating the expression of AtWRKY23 (Grunewald et al., 2012). AtWRKY22 positively regulates senescence and can influence the expression of its own gene and of *AtWRKY53* and *AtWRKY70* (Zhou et al., 2011). AtWRKY70 functions downstream of SNC2–1D (suppressor of npr1–1, constitutive 2) and regulates plant immunity (Zhang et al., 2010). At post transcriptional level miRNAs can regulate the expression of various WRKY TFs to modulate various processes. As shown in **Figure 2**, miR396 regulates high temperature response in sunflower through HaWRKY6 (Giacomelli et al., 2012). Stress responsive WRKYs are tightly regulated during normal condition but after occurrence of a stress particular signal is transmitted that lead to activation of the responsive TFs.

### Regulation by Kinases

MAP Kinases (MPK) play an important role in the activation of WRKYs to regulate various responses (Adachi et al., 2015). AtWRKY33 forms a complex with MAP kinase MPK4 in the nucleus. Upon triggered by MAMP or PAMP perception MPK, MKK (MAP kinase kinase) and MEKK (MAP kinase kinase kinase) are activated, which leads to nuclear dissociation of the MPK4–MKS1–WRKY33 complex releasing AtWRKY33 and MKS1. Then AtWRKY33 activates PAD3 (phytoalexin deficient 3) that is required for antimicrobial camalexin synthesis (Qiu et al., 2008). During post-association with MKS1 (MAPK substrate1), VQ protein interacts with AtWRKY33/25 to act as a substrate of MAPK4 (Qiu et al., 2008). Also MEKK1 directly binds to the WP1 region in the promoter of *AtWRKY53* that is present upstream of a W box where AtWRKY53 itself binds to its promoter. It leads to phosphorylation of AtWRKY53 by MEKK1 that increases the binding affinity of AtWRKY53 to its own promoter (Miao et al., 2007). Activation domain protein (AD protein) also interacts with the promoter of *AtWRKY53*. Actually both MEKK1 and AD protein physically interacts with each other and enhances *AtWRKY53* expression. However MEKK1 does not phosphorylate AD protein, which can phosphorylate itself (Miao et al., 2008). Many WRKYs like AtWRKY28 are substrates of calcium-dependent protein kinases like CPK4 and CPK11 (Gao and He, 2013). *P. syringae* pv. Tomato DC3000 infection induces expression of PKS2 (SOS2-like protein kinase 5) that interacts with the AKR (Ankyrin Repeats) motif and phosphorylates NPR1 (Non-expressor of Pathogenesis-Related gene 1). This interaction results in the upregulation of *AtWRKY38* and *AtWRKY62* involved in mediating plant defense responses (Xie et al., 2010). OsWRKY53 suppresses herbivore-induced defense in rice by negative feedback modulation of MPK3/MPK6 activity (Hu et al., 2015). MAPK4 interacts with MKS1, which in turn interacts with AtWRKY25 and AtWRKY33 to act as a negative regulator of SA-mediated defense responses to *P. syringae* (Andreasson et al., 2005). When *M. oryzae* or *Xanthomonas oryzae pv oryzae* attacks rice, MAPK regulates expression of OsWRKY45 and provides resistance to these infection by SA/benzothiadiazole (BTH)-mediated defense (Matsushita et al., 2012; Nakayama et al., 2013). AtWRKY22 and AtWRKY29 are involved in MAPK pathway regulated responses to both bacterial and fungal pathogens (Göhre et al., 2012). Therefore phosphorylation and activation by kinases is an important regulatory mechanism that can be targeted for controlled expression of certain TFs.

### Regulation by Epigenetic Mode

Non-genetic influence of WRKY gene expression is also seen which deeply affect several physiological responses. ATX1 (Trithorax) activates the expression of *AtWRKY70* epigenetically. They leads to nucleosomal histone H3K4 trimethylations that activates *AtWRKY70*, which in turn activates PR-1 and THI2.1 defense genes (Alvarez-Venegas et al., 2007). SUVH2 histone methyltransferase leads to H3K4me2 and H3K4me3 methylation that epigenetically regulates *AtWRKY53* to mediate leaf senescence responses (Ay et al., 2009). Histone Deacetylase 19 (HDA19) represses transcription of *AtWRKY38* and *AtWRKY62* by removing acetyl groups from histone tails and thus negatively regulates basal defense (Kim et al., 2008). Histone methylations at the *AtWRKY40* promoter activate the SA-dependent pathway to control plant immunity (Alvarez et al., 2010). Also histone methylation at *AtWRKY40* promoter inhibits expression of *ABI5* and negatively regulates ABA signaling in seed germination and post-germination growth (Shang et al., 2010). FLD (flowering locus D- a homolog of human-lysine-specific histone demethylase) epigenetically influences systemic-acquired-resistance induced expression of *AtWRKY29* and *AtWRKY6* through histone modifications at their promoters (Singh et al., 2014). Linker histone H1 gene MaHIS1 interacts with *MaWRKY1* to regulate physiological processes like ripening and stress responses in banana fruit (Wang et al., 2012). As discussed earlier WRKYs tend to interact with different VQ proteins, which have been hypothesized to induce histone modification and chromatin remodeling to regulate downstream genes (Lai et al., 2011). Whether it is covalent modifications, structural inheritance or nucleosome positioning, gene expression and downstream translation is dependent on theses factors. Therefore these epigenetic mode of regulations need to be addressed before turning to genetic way of modifications.

### Regulation by Proteasome System

Expression level of stress responsive WRKYs under normal condition are kept under check by different mechanisms one of which is proteasome-mediated degradation. As stated above OsWRKY45 plays a major role in SA/BTH induced defense, which is actually regulated through nuclear UPS (ubiquitin proteasome system). Under normal condition UPS rapidly degrades OsWRKY45 in nuclei in order to suppress defense responses but onset of pathogen attack inhibits proteasomes and induces accumulation of polyubiquitinated OsWRKY45 (Matsushita et al., 2013). The domains required for UPS-dependent degradation lie closely to the transactivation domain of OsWRKY45 (Matsushita et al., 2013). AtWRKY53 that positively regulates pathogen response negatively regulates leaf senescence. HECT domain E3 ubiquitin ligase UPL5 (Ubiquitin protein ligase 5) interacts with AtWRKY53 via its leucine zipper domain for its polyubiquitination and degradation. AtWRKY53 expression is tightly regulated as to induce pathogen response or senescence in the proper time frame (Miao and Zentgraf, 2010). Ubiquitin-mediated regulation of WRKYs is also observed in *Vitis pseudoreticulata* for positive regulation of defense responses to pathogen attack. EIRP1 (E3 ubiquitin ligase Erysiphenecator-induced RING finger protein 1) interacts with VpWRKY11 through the RING domain. EIRP1 mediates proteolysis of VpWRKY11 *via* degradation by the 26S proteasome and inhibits W-box-dependent transcription (Yu et al., 2013). Proteasome mediated regulation of various WRKYs at normal condition and their activation by kinases under stress is shown in **Figure 2**. So this aspect is also necessary to identify the entire network involved in stress response and very limited information is available at this time. Particular attention is required in the field of other crops so that stress tolerance and susceptibility issues could be attended.

### Regulation by Retrograde Mechanism

**FIGURE 3:**
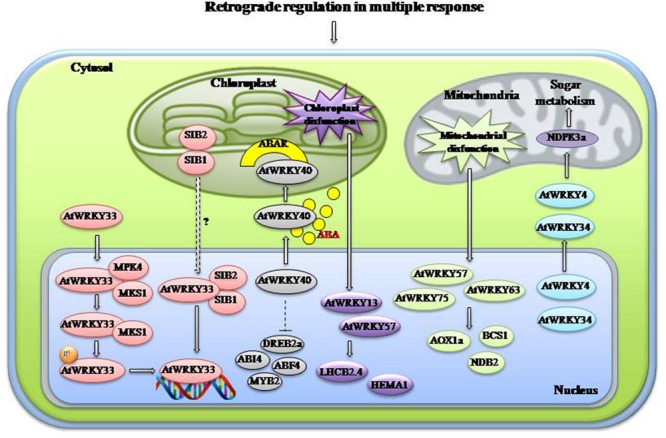
**Retrograde regulation in multiple responses.** WRKYs play an imortant role in organellar interactions. During plastid disfunction AtWRKY13/40/57 are activated which regulate chloroplast associated genes like LHCB2.4 and HEMA1. Under normal condition AtWRKY40 represses expression of stress-responsive genes like DREB2a, ABI4, ABF4, and MYB2. When ABA is accumulated under stress it leads to translocation of AtWRKY40 to the chloroplst membrane located protein ABAR so that expression of stress-responsive genes could be elevated. Chloroplst targeted proteins SIB1/2 help in the activation and binding of AtWRKY33 to the promoter of downstream genes with the help MAPK4 and MKS1. Mitochondrial disfunction also activates certain WRKYs like AtWRKY57/63/75 which in turn activates mitochondria associated genes AOX1a, BCS1, and NDB2. AtWRKY4/34 regulate NDPK3a and sugar metabolism probably through mitochondrial retrograde signaling. Abbreviations: ABAR, ABA receptor; NDPK3a, Nucleoside diphosphate kinase 3a; ABF4, ABRE-binding factor 4; MPK4, Mitogen activated protein kinase 4; MKS1, MAP kinase substrate 1; SIB, Sigma factor binding protein; ABI4, ABA insensitive 4; LHCB, Photosystem II chlorophyll a/b-binding polypeptide gene; AOX1a, Alternative oxidase 1a; NDB2, type II NAD(P)H dehydrogenases B2.

WRKYs are also regulated through inter-organelle retrograde signaling. One example of chloroplast-mediated retrograde regulation revolves around AtWRKY18-40-60 cluster. AtWRKY18, AtWRKY40, and AtWRKY60 act as negative regulators of ABA signaling, inhibiting seed germination and post-germination growth. They interact with cytosolic C-terminus of ABAR (magnesium-protoporphyrin IX chelatase H subunit that function as an ABA receptor) that is located in the chloroplast envelope. AtWRKY40 acts as a central negative regulator that inhibits expression of ABA-responsive genes like ABI4, ABI5, and ABF4. In presence of high level of ABA, AtWRKY40 is recruited in the cytosol from nucleus that promotes ABAR-AtWRKY40 interaction. ABAR represses expression of AtWRKY40 and allow expression of ABA responsive genes (Shang et al., 2010). An example of mitochondrial retrograde regulation involves plant NDPKs (nucleoside diphosphate kinases) that are involved in stress, hormone response, and light signaling (Hammargren et al., 2008). NDPK3a is located in mitochondria and carry 2 WBOXHWISO1 boxes in its promoter that are involved in sugar metabolism and signaling. SUSIBA2 (HvWRKY46), AtWRKY4 and AtWRKY34 are associated with sugar induction and expression of NDPK3a (Pesaresi et al., 2007; Hammargren et al., 2008). As mentioned earlier AtWRKY53 has diverse roles and its expression is tightly regulated (Sun and Yu, 2015). AD protein, which physically interacts with AtWRKY53 and induces its expression, is also located in plastids (Miao et al., 2008). Whirly1 a dual-targeted protein too regulates AtWRKY53 expression. Nuclear isoform of Whirly1 acts as an upstream regulator and directly represses the expression of AtWRKY53 during un-intended leaf senescence. Whirly1 interacts with the elicitor response element motif-like sequence (GNNNAAATT) and an AT-rich telomeric repeat-like sequence present in the promoter of *AtWRKY53* suppressing its expression and of downstream genes like *AtWRKY33, SAG101*, and *SAG12* involved in senescence. The plastid form of Whirly1 is involved in positive regulation of AtWRKY53 in nucleus through retrograde signaling (Miao and Zentgraf, 2010; Miao et al., 2013). AtWRKY40 acts as a repressor of high-light-induced signaling and antimycin A-induced mitochondrial retrograde expression while AtWRKY63 acts as an activator. They are involved in the regulation of stress-responsive organelle proteins which are responsive to both mitochondrial and chloroplast dysfunction (Van Aken et al., 2013). AtWRKY57, AtWRKY63, and AtWRKY75 show stress response by regulating nuclear encoded organelle proteins like *AOX1a* (Van Aken et al., 2013). AtWRKY15 leads to expression of genes involved in mitochondrial dysfunction regulon and negatively regulates retrograde signaling (Vanderauwera et al., 2012). Interaction of WRKYs with VQ proteins have been assumed to play roles in regulation of transcription and retrograde signaling from chloroplast/mitochondria to the nucleus (Lai et al., 2011). Retrograde signaling of different WRKYs operating from chloroplast and mitochondria to nucleus is shown in **Figure 3**. Anterograde and retrograde mechanism are dependent on each other and this signaling has many lacunas that need to be filled prior development of genetically modified plants.

## Crop Improvement by Regulating Multiple Responses and/or Traits Through WRKYs

Plants being non-motile are susceptible to various environmental factors. As stated above they can be engineered to overcome that susceptibility through controlled regulation of TFs like WRKY. They display multiple, interconnected, complex, and flexible expression patterns in response to various stimuli which could be exploited to obtain varieties that are resistant and tolerant to environmental fluctuations. Also complete control over an entire network by modulation of a single gene responsive to a particular action is not possible. But TFs like WRKY can regulate multiple sets of genes involved in an interconnected network cooperatively and simultaneously. Initiation of crop improvement through transgenic approach is still in its early phase. But certain productive steps have been taken to ensure success in long term projects.

### Rice

It is the most widely consumed staple food for a large part of the world’s human population as a cereal grain. Also it is a model plant where substantial research has been done, which can be exploited for generation of improved breeds with very limited unwanted traits. OsWRKY11 under control of HSP101 promoter leads to more chlorophyll content and low leaf wilting in rice under drought stress (Wu et al., 2009). OsWRKY30/47 overexpressed transgenic rice displayed drought tolerance (Shen et al., 2012; Raineri et al., 2015b). Rice with overexpressed OsWRKY76 showed susceptibility to pathogens while tolerance to cold stress (Yokotani et al., 2013). OsWRKY89 overexpressed rice plants showed increased wax deposition leading to UV-B tolerance and disease resistance (Wang et al., 2007). OsWRKY4 mediates defense responses toward *Rhizoctonia solani* in rice (Wang H. et al., 2015). OsWRKY6 positively regulates defense response in overexpressed rice (Choi et al., 2015). OsWRKY53 regulates herbivore-induced defense responses in rice (Hu et al., 2016). In rice OsWRKY24/45 negatively and OsWRKY72/77 positively regulates an ABA-inducible promoter which can be engineered to promote abiotic stress response (Xie et al., 2005). Another WRKY from rice OsWRKY74, modulates tolerance to phosphate starvation (Dai et al., 2016). Modulation of *OsWRKY4* transcript levels by constitutive overexpression in rice increases resistance to the necrotrophic sheath blight fungus *Rhizoctonia solani* (Wang H. et al., 2015). OsWRKY6 interacts with the promoter of *OsPR10a* and *OsICS1* providing enhanced disease resistance to pathogens in rice (Choi et al., 2015). Two transcriptional repressors OsWRKY13 and OsWRKY42 and activator WRKY45-2 forms a transcriptional regulatory cascade that provides resistance to fungal pathogen *M. oryzae* (Cheng et al., 2015). So there are potential TFs that can enhance the tolerance of rice towards different stresses but upstream and downstream components needs to be carefully studied for transgenic development.

### Others

Overexpression in homologous system is a difficult task as transformation and tissue regeneration protocols differ from species to species. Nevertheless there are few examples where WRKYs have been successfully overexpressed in their native host and found some promising results. CmWRKY17 when overexpressed in *Arabidopsis* and *Chrysanthemum* showed reduced salt stress tolerance (Li et al., 2011, 2015b). CmWRKY15 facilitated *Alternaria tenuissima* infection by antagonistically regulating the expression of ABA-responsive genes while CmWRKY48 enhanced aphid resistance in transgenic *Chrysanthemum* (Fan et al., 2015; Li et al., 2015a). CmWRKY1 leads to dehydration tolerance in *Chrysanthemum* by regulating ABA-associated genes (Fan et al., 2016). PtrWRKY19 when overexpressed in *Populus trichocarpa*, negatively regulates secondary cell wall formation in pith parenchyma cells (Yang L. et al., 2016). Overexpression of PtoWRKY60 in poplar resulted in increased resistance to *Dothiorella gregaria* (Ye et al., 2014). ThVHAc1 regulated by ThWRKY7 provides cadmium stress tolerance in *Tamarix hispida* (Yang G. et al., 2016). CaWRKY6 regulates the expression of CaWRKY40, confers resistance to *R. solanacearum* infection, and provides tolerance to high-temperature and high-humidity in pepper (Cai et al., 2015). GmWRKY27 interacts with GmMYB174 to reduce expression of GmNAC29 that leads to stress tolerance in soybean plants (Wang F. et al., 2015). Since these TFs play an important role in increasing stress tolerance and developmental responses in plants, they can be targeted for generation of improved varieties using transgenic technology. Still large numbers of WRKY transcripts are uncharacterized in *Arabidopsis* and rice, and many more in other plants. Therefore much more exploration and proper study is required to fully understand the WRKY governed plant responses. Recent advancement in technology would be helpful in analysis of these unanswered questions.

## Conclusion and Future Perspective

Proper growth and development of plants is critically dependent on surrounding environmental conditions. Many times abiotic stresses are accompanied by biotic stresses. In this review we brought up the fact that TFs like WRKY can regulate genes involved in multiple responses at the same time. This aspect carries potential benefit for the economically important plants. Transgenic approach though in its early infancy could be targeted for development of plants with tolerance to multiple stresses. WRKYs not only regulate stress and developmental responses they are also involved in specialized metabolic pathways. We also observed that WRKYs themselves are regulated tightly to maintain normal cellular homeostasis under normal condition. Through genetic alteration and recombinant technology, traits regulated by WRKYs could be modulated for development of better varieties. We strongly believe that ‘regulation of WRKY’ and ‘regulation by WRKY’ should be explored in detail; and particular attention should be given to the following points to understand the entire crosstalk and cascade involved in multiple responses by a single TF:

(1) In transgenics along with WRKYs proper regulation of promoters should be done. Also post-transcriptional and translational changes should be monitored to remove or minimize the negative unwanted effects. With it, orthologs and homologs should be studied that would identify the precursors or substrates involved in different regulatory cascades under stressed condition.
(2) Study of signaling molecules, interacting partners and phosphorylating agents is necessary. Further the TF-dependent integrated web in which different other TFs like ERF, MYB, MYC, and NAC act along with WRKY to regulate various responses, needs to be studied.
(3) Through the advancement in high-throughput transcriptomic, proteomic, metabolomic platforms analysis of large set of data is quite fisible. These technologies in association with microarray would help us to understand the diverse WRKY-associated networks. Novel development and stress-responsive TFs could be identified which have naturally adapted to hazardous environmental conditions.
(4) CRISPR (Clustered Regularly Interspaced Short Palindromic Repeats) and CRISPR-associated (Cas) gene system could be an effective way to study functional aspect of WRKYs. Traditional silencing or VIGS could not entirely mask the expression of target genes. The type II CRISPR mechanism involving Cas9 could be targeted to generate potential functional mutants in non-model plants. So to elucidate and interpret the role of WRKYs, not only overexpression but also silencing of the concerned gene is necessary which could be attained by CRISPR/Cas9 strategy in near future. Also negative regulatory WRKY genes could be manipulated for better response in terms of stress tolerance or better yield.
(5) One major hinderence in the transgenic approach is the long term field trials which has some issues with horizontal gene transfer and unwanted characters. So proper field trials and accurate carefull observation of modified traits should be monitored. Commercialization of genetically modified crops should be promoted with thorough study and precaution. These mentioned aspects would give a deeper understanding of the plant stress responses and allow plants of agricultural and commercial importance to survive under environmental fluctuations.

## Author Contributions

RS has conceptualized the theme of review. UP has prepared the first draft after collecting literature. GJ has added the work related to secondary metabolism. Finally both UP and GJ have compiled it. RS has edited the manuscript and finalized the draft. All three authors have revised the MS.

## Conflict of Interest Statement

The authors declare that the research was conducted in the absence of any commercial or financial relationships that could be construed as a potential conflict of interest.
